# The need for larval source management accompanying urban development projects in malaria endemic areas: a case study on Bioko Island

**DOI:** 10.1186/s12936-022-04362-9

**Published:** 2022-11-14

**Authors:** Guillermo A. García, Godwin Fuseini, Olivier Tresor Donfack, Rachel N. Wofford, Jose Antonio Mba Nlang, Prudencio Bibang Efiri, Valeriano Oluy Nsue Maye, Thomas A. Weppelmann, David Galick, Wonder Philip Phiri, Kylie DeBoer, Jordan M. Smith, Jeremias Nzamio Mba Eyono, Matilde Riloha Rivas, Carlos A. Guerra, Michael E. von Fricken

**Affiliations:** 1 MCD Global Health, Bioko Island Malaria Elimination Project, Silver Spring, MD USA; 2 MCD Global Health, Bioko Island Malaria Elimination Project, Bioko Island, Equatorial Guinea; 3 Department of Global and Community Health, George Mason University, Fairfax, VA USA; 4 Department of Internal Medicine, Morsani College of Medicine, University of South Florida, Tampa, FL USA; 5 Ministry of Health and Social Welfare of Equatorial Guinea, National Malaria Control Programme, Malabo, Equatorial Guinea

**Keywords:** Larval source management, Malaria, Anopheles, Construction, Larval habitats

## Abstract

**Background:**

In 2017, several new housing districts were constructed on Bioko Island, Equatorial Guinea. This case study assessed the impact construction projects had on mosquito larval habitats and the effectiveness of larval source management in reducing malaria vector density within the surrounding area.

**Methods:**

*Anopheline* larval presence was assessed at 11 new construction sites by the proportion of larval habitats containing *Anopheline* pupae and late instar larval stages. *Bacillus thuringiensis israelensis* (Bti) larvicide was applied weekly to nine locations for 30 weeks, while two locations received no larvicide and acted as controls. Adult mosquito density was monitored via human landing collections in adjacent communities of six construction sites, including the two control sites.

**Results:**

The sites that received Bti had significantly lower observation rates of both pupae (3.2% vs. 18.0%; p < 0.001) and late instar *Anopheles* spp. mosquitoes (14.1 vs. 43.6%; p < 0.001) compared to the two untreated sites. *Anopheles* spp. accounted for 67% of mosquitoes collected with human landing collections and were captured at significantly lower levels in communities adjacent to treated construction sites compared to untreated sites (p < 0.001), with an estimated 38% reduction in human biting rate (IRR: 0.62, 95% CI IRR: 0.55, 0.69). Seven months after the start of the study, untreated sites were treated due to ethical concerns given results from treatment sties, necessitating immediate Bti application. The following week, the number of habitats, the proportion of larval sites with *Anopheles* spp. pupae, late instars, and adult biting rates in adjacent communities to these sites all decreased to comparable levels across all sites.

**Conclusion:**

Findings suggest larval source management represents an effective intervention to suppress mosquito populations during infrastructure development. Incorporating larval source management into ongoing and planned construction initiatives represents an opportunity to fine tune vector control in response to anthropogenetic changes. Ideally, this should become standard practice in malaria-endemic regions in order to reduce viable mosquito habitats that are common by-products of construction.

**Supplementary Information:**

The online version contains supplementary material available at 10.1186/s12936-022-04362-9.

## Background

Bioko Island, Equatorial Guinea, has seen dramatic reductions in malaria transmission over the past 18 years thanks to intensive vector control interventions [1]. Two of the main malaria vectors, *An. gambiae *sensu stricto and *An. funestus*, were eliminated relatively soon after introducing island-wide indoor residual spraying [2–4], but populations of *An. coluzzii* and *An. melas* remain resilient to current interventions and are responsible for recent malaria resurgence [5]. These vector populations maintain a high malaria transmission potential on the island that stands as a reminder of pre-intervention times when local annual entomological inoculation rate estimates surpassed one thousand infective bites per person in some sites [6].

Species of the *An. gambiae* complex typically oviposit in a wide range of water bodies, including swamps, rice fields, wells, ponds, puddles, hoof prints, discarded tin cans, and both permanent and temporary water collection bins. The creation of anopheline larval habitats is often linked to anthropogenic land-use changes, chiefly deforestation and agricultural expansion, that can undermine malaria control and elimination efforts [5, 7, 8]. While urbanization is another major cause of anthropogenic land-use change, most anopheline mosquito populations decline in urban environments, consequently reducing malaria transmission in large urban agglomerations [9]. However, urban growth in malaria-endemic settings often combines rapid deforestation and the generation of temporary water collections that prove ideal for anopheline mosquito oviposition.

Larval source management (LSM) targets the immature mosquito larvae and pupae by killing them at the source (larval site), eliminating the aquatic habitats, or making these unsuitable for mosquito oviposition [10]. The contribution of LSM to malaria vector control has been contentious and recommended as complementary in specific situations, though it has also been regarded as a potentially key intervention by the World Health Organization (WHO) [10] for integrated vector management and advocated as part of the Roll Back Malaria expansion of the vector control toolbox. Larval source management is beneficial because, unlike household interventions, it tackles both indoor and outdoor mosquito biting by reducing vector densities [11]. One standard method of LSM is the use of *Bacillus thuringiensis israelensis* (Bti), a bacterium species that kills mosquito larvae by releasing a gut paralyzing toxin, causing starvation [12]. The main advantages of Bti are that it is relatively inexpensive, easy to implement, and environmentally safe. Bti is regularly used on Bioko Island to complement the core vector control interventions, indoor residual spraying (IRS), and LLINs and is viewed as an important component in combating emerging insecticide resistance [13].

This study examines the impact of Bti based LSM on anopheline mosquito larvae and pupae populations observed in anthropogenic larval habitats generated by urban development projects on Bioko Island, with data matched to adult mosquito densities near treated and untreated sites.

## Methods

### Study sites

**Fig. 1:**
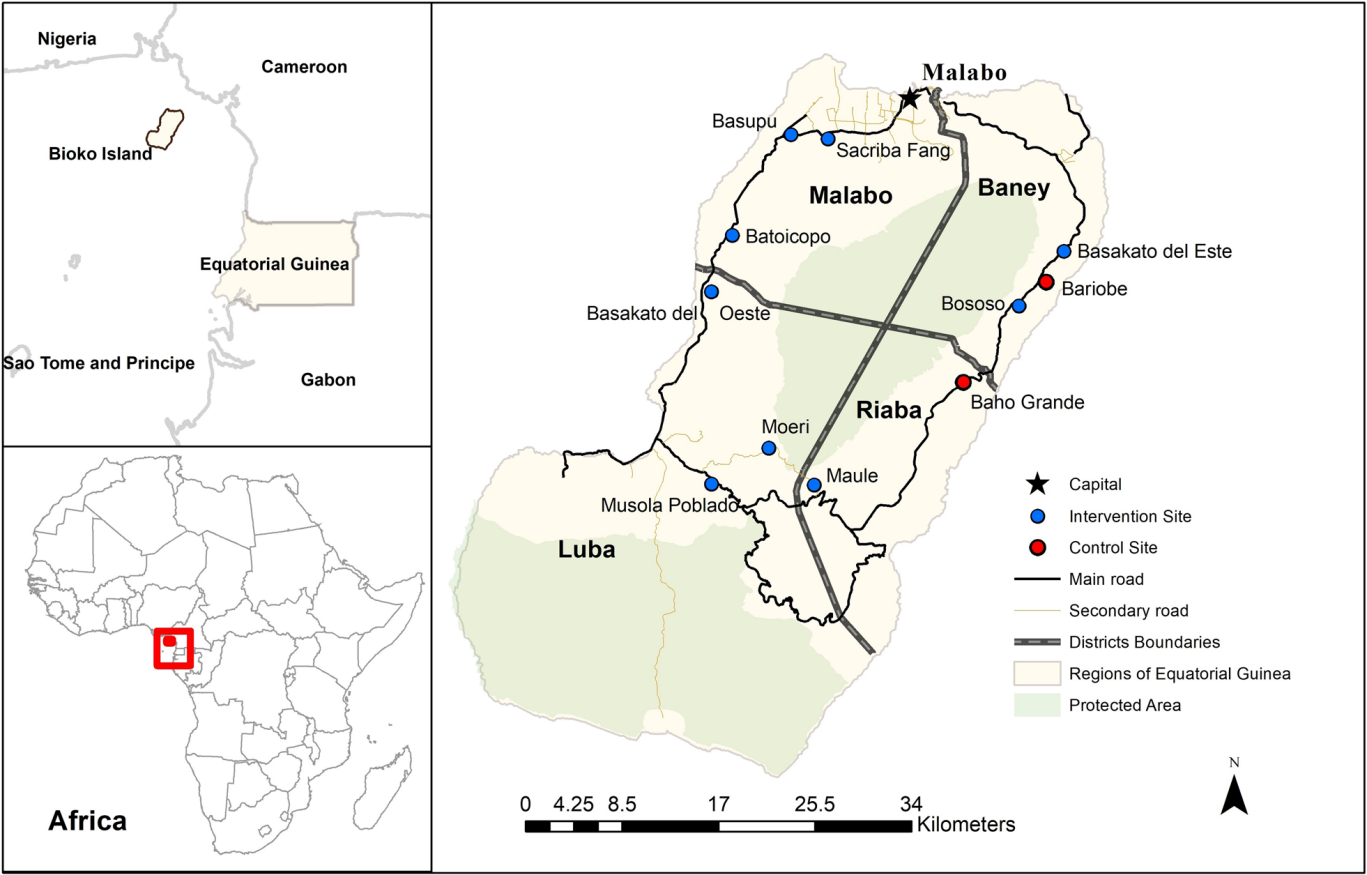
Map of Bioko Island, Equatorial Guinea, with study locations. A map of Bioko Island, Equatorial Guinea, is presented with the geographic relation to the region of West Central Africa and the African continent, along with the capital city of Malabo, major roadways, and administrative districts. The monitored construction sites are also presented, with red circles representing control sites (no larvicide) and blue circles representing intervention sites (larvicide applied)

Bioko Island is the main island of the Insular Region of Equatorial Guinea and is located in the Gulf of Guinea, some 40 km off the coast of Cameroon (Fig. 1). Malaria transmission is perennial, with hot and humid conditions favourable for vector proliferation and parasite development, though transmission increases in the months of June to December when rainfall peaks. This study was conducted between May and December 2017, spanning 30 weeks during the construction of 11 peri-urban housing projects across Bioko Island (Fig. 1). Construction lots ranged in size from 120 to 300sqm, with an average size of ~ 200sqm. All construction started at roughly the same time as part of an urbanization effort of rural communities, carried out by the government of Equatorial Guinea.

### Identification of larval sites and larvicide application

Potential mosquito larval habitats were sought and mapped weekly at all 11 construction sites. Data on habitats were reassessed upon each visit, given changes in landscape features due to ongoing construction. Recorded data included the habitat type (*i.e.* drainage gutters/canals, indentation from construction machinery (wheel prints), puddles, water containers, and ‘other’ sources), the approximate area of the habitat, in square meters [*i.e.* small (< 10 m^2^), medium (10 to 100 m^2^) and large habitats (> 100 m^2^)], the presence of late-stage anopheline larvae (instars 3 and 4) and pupae. Larval habitats were considered ‘positive’ if larvae or pupae were observed, regardless of density. At nine of the sites, all identified habitats received an application of the Bti, hereafter referred to as *treated*; two sites did not receive such treatment, hereafter referred to as *untreated*, which were selected randomly from the list of all active construction sites. Additional image files show the environment during and post-construction, including the LSM team working an active construction site and the completed construction project (see Additional files 1 and 2). The same entomology teams were used to identify, categorize, and treat potential larval sites, which allowed for standardization in data collection. All identified larval habitats were by-products of construction activity and were contained within the perimeters of the construction sites, with no treatment of the surrounding areas, which were primarily classified as jungle landscape.

### Human landing catches

Human landing catches (HLC) were conducted once a month in three houses located within 100 m of six sites, including four where habitats were treated and two sites where habitats were left untreated. Teams of four collectors per house, two indoors and two outdoors, worked between 19h00 and 6h00, rotating their positions at midnight. Workers caught mosquitoes that landed on their exposed arms and legs. These samples were enumerated and later morphologically identified. From these landings, the human biting rate (HBR) was estimated and measured as the number of bites per person per hour (b/p/h). The risks involved with the study were conveyed to HLC collectors, and free diagnosis and treatment was provided to any individual who showed symptoms [14]. Ethical approval for this study was granted by the National Malaria Control Programme (NMCP) of the Ministry of Health and Social Welfare, Equatorial Guinea. The housing structures where HLC occurred were primarily low income and shared the same characteristics (i.e., wooden structure, no screened windows, open eaves). Prior to initiating HLC, volunteer homes were assessed for consistency across sites.

### Rainfall data

Rainfall data (inches per week) from May 1st to December 31st, 2017, were obtained from the NOAA Global Summary of Day (GSOD), collected from the Bioko Island, Malabo International Airport weather station. This data was used as a proxy for island-wide rainfall across sites, since alternative data sources were not available to monitor site specific precipitation.

### Statistical analyses

The primary outcomes of the study were the presence of aquatic habitats with *Anopheles* spp. larvae and pupae, and the anopheline density and HBR in neighboring communities. The likelihood of detecting pupae and late larvae instars was determined using logistic regression. Predictors were set as sites with and without Bti, and stratification was conducted for each type and size of the larval site. The HBR was compared between sites using a Poisson regression model, with and without adjustment for site characteristics and study week. All statistical analyses used an alpha value of 0.05 to determine statistical significance and were conducted using STATA software version 15 (StataCorp, College Station, Texas, USA).

## Results

### Habitat characterization and distribution

**Fig. 2:**
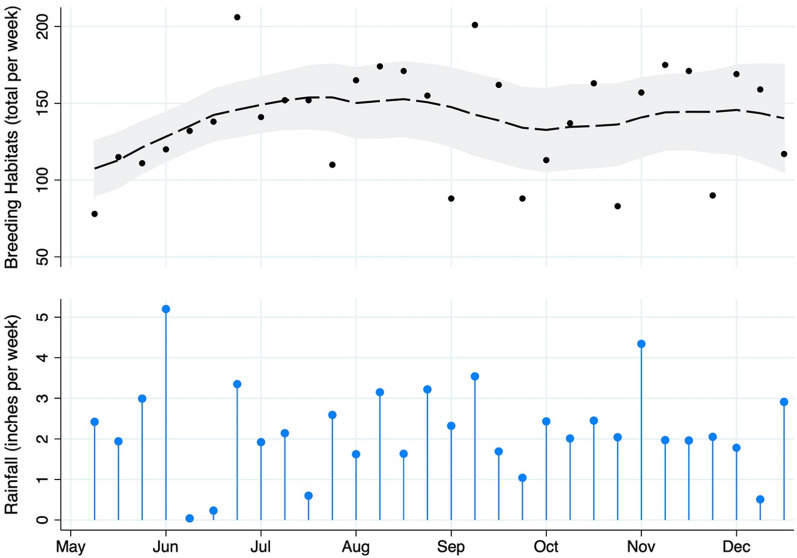
Total Number of Larval Habitats Identified and Rainfall. The number of potential larval habitats identified at the construction sites are presented with respect to study week (May 1, 2017, is week one), with 95% confidence intervals depicted in gray, and the total weekly rainfall in inches

Throughout the study, 4,197 potential larval habitats were identified, 3453 at the nine construction sites where Bti larvicide was used (mean of 384 per site) and 744 at the two sites that received no Bti larvicide (mean of 372 per site). The number of potential larval sites increased with increasing rainfall at the beginning of the study period and fluctuated between < 100 and 200 per week (~ 150/week) until December (Fig. 2). Details on habitats identified, treatment status, and size can be found in Table 1. The majority of habitats were water gutters/canals (60.7%), followed by wheel imprints (22.2%). Most were medium-sized (10–100 m 65.2%) though a few were of considerable size (> 100 m^2^; 9.3%).

**Table 1:** Characteristics of potential larval habitats

Potential larval habitat characteristics						
Characteristic	Total		Untreated		Treated	
Type	*n*	*%*	*n*	*%*	*n*	*%*
Gutters/Canals	2548	60.7	283	38.0	2265	65.6
Wheel prints	930	22.2	215	28.9	715	20.7
Puddles	470	11.2	191	25.7	279	8.1
Water container	142	3.4	55	7.4	87	2.5
Other	107	2.6	0	0.0	107	3.1
Size						
< 10m^2^	986	23.5	353	47.5	633	18.3
10 to 100m^2^	2737	65.2	297	39.9	2440	70.7
> 100m^2^	391	9.3	61	8.2	330	9.6
Undetermined	83	2.0	33	4.4	50	1.4
Total	4197		744		3453	

The number and proportion of potential mosquito larval habitats are presented by the type of site and the size of the larval site (area in metres) for all construction sites and separately for control and intervention sites. A small proportion of the habitats could not be classified into larger categories (other), or the size was not accurately determined (undetermined)

### Presence of *Anopheles* pupae and late instars

**Fig. 3:**
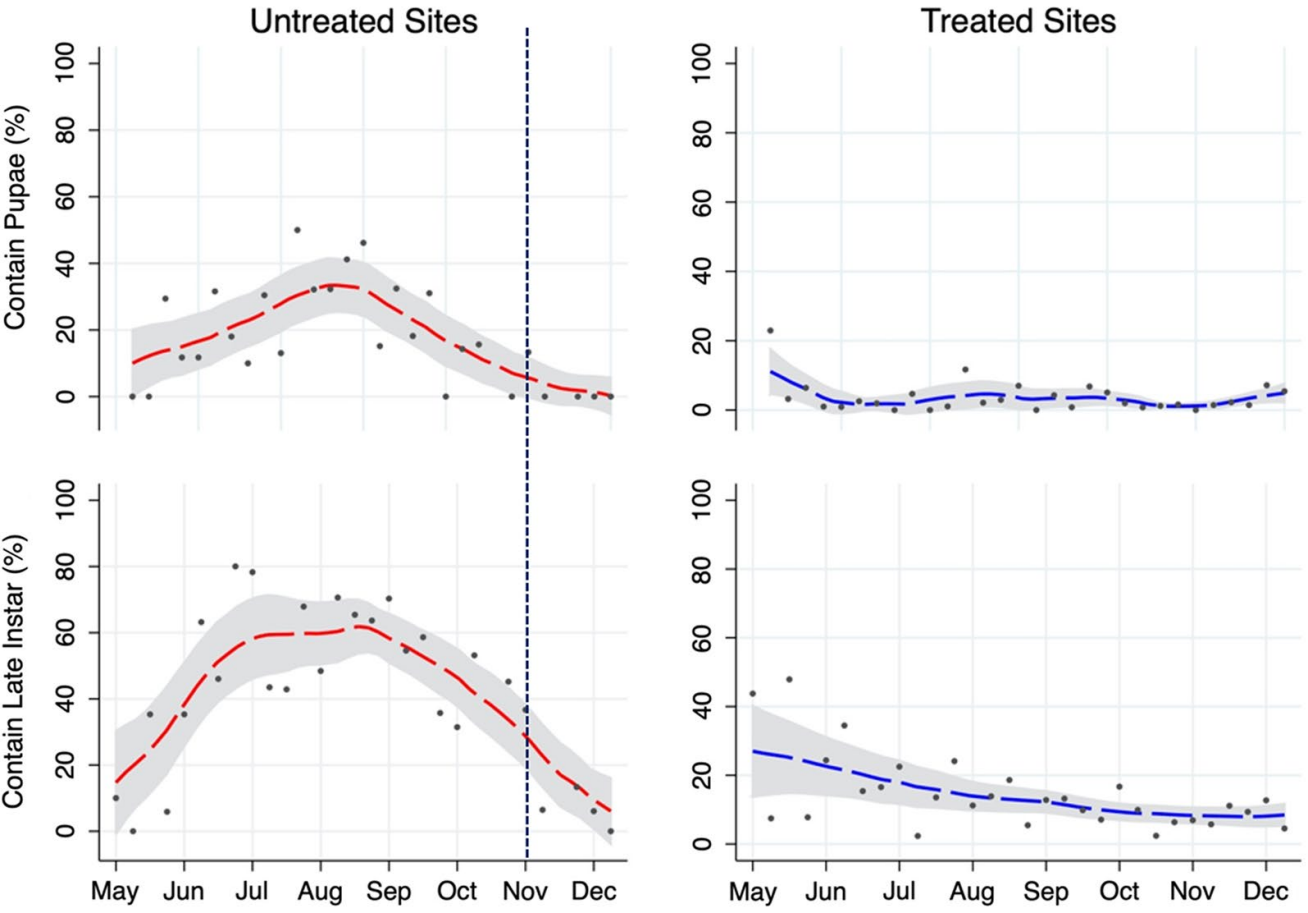
The proportion of habitats with pupae and larvae for *Anopheles* spp. mosquitoes. The proportion of potential habitats that contained mosquito pupae or late instar larvae are presented with respect to study week (May 1, 2017, is week one) for untreated (red, left side) and LSM treated (blue, right side) construction sites. Both the actual average proportions (black dots) and smoothed average (dotted line) are shown, along with a 95% confidence interval for the smoothed average (gray shading). The vertical purple dash line indicates when the untreated sites were treated as a public health precaution due to increased adult mosquito activity within the surrounding area

The proportion of habitats with pupae or late instar larvae is presented for both treated and untreated construction sites by study week and habitat characteristics (Tables 2 and 3, and Fig. 3). In untreated sites, there were significant differences in the presence of pupae by type (p = 0.006) and size (p < 0.001) of habitat, with water containers having the lowest proportion of observed detections (3.6%) and larval habitats over 100 m^2^ having the highest frequency of pupae observations (36.1%). There was no significant difference in the habitat positivity rate in the treated sites by type (p = 0.08) or size (p = 0.06). Treated sites had a significantly lower presence of pupae compared to untreated sites (3.2% vs. 18.0%; p < 0.001), with an estimated reduction of 85% (OR: 0.15; 95% CI OR: 0.12, 0.2). This difference was significant for all habitat types and sizes (p < 0.002), excluding water containers (p = 0.341). In addition, Bti treated sites had a significantly lower presence of late instars compared to untreated sites (14.1 vs. 43.6%; p < 0.001), with an estimated reduction of 79% (OR: 0.21; 95% CI OR: 0.18, 0.25), with this difference observed in all habitat types and sizes (p < 0.001).

**Table 2:** Presence of *Anopheles* spp. pupae in potential larval habitats for treated and untreated sites

Presence of pupae in potential larval habitats							
Characteristic	Untreated		Treated		Comparison		
Type	*obs*	*% pos*	*obs*	*% pos*	*p*	*OR*	*95% CI*
Gutters/Canals	283	19.8	2265	3.5	< *0.001*	0.15	0.10, 0.22
Wheel prints	215	22.8	715	2.9	< *0.001*	0.10	0.06, 0.18
Puddles	191	14.1	279	2.5	< *0.002*	0.16	0.07, 0.37
Water container	55	3.6	87	1.1	0.341	0.31	0.03, 3.48
Other	0	0.0	107			1.00	
Size							
< 10m^2^	353	14.7	633	2.4	< *0.001*	0.14	0.08, 0.25
10 to 100m^2^	297	20.2	2437	2.8	< *0.001*	0.11	0.08, 0.15
> 100m^2^	61	36.1	330	4.8	< *0.001*	0.09	0.04, 0.19
Undetermined	33		50			1.00	
Total	744	18.0	3453	3.2	< *0.001*	0.15	0.12, 0.20

The number of observations and proportion of potential mosquito larval habitats containing pupae are presented by habitat type and size for control and intervention sites with statistical comparison using logistic regression (significant values appear in italics). A small number of habitats that were not classified into larger groups or the size was not able to be determined were not included in the above statistical comparison

**Table 3:** Presence of *Anopheles* spp. late-stage larvae in potential larval habitats for control and intervention sites

Characteristic	Untreated		Treated		Comparison		
Type	*obs*	*% pos*	*obs*	*% pos*	*p*	*OR*	*95% CI*
Gutters/Canals	283	37.8	2265	15.3	< *0.001*	0.30	0.23, 0.39
Wheel prints	215	52.1	715	13.8	< *0.001*	0.15	0.11, 0.21
Puddles	191	47.1	279	11.5	< *0.001*	0.15	0.09, 0.23
Water container	55	27.3	87	5.7	*0.001*	0.16	0.06, 0.50
Other	0		107				
Size							
< 10m^2^	353	42.5	633	11.4	< *0.001*	0.17	0.13, 0.24
10 to 100m^2^	297	45.1	2,440	13.9	< *0.001*	0.20	0.15, 0.25
> 100m^2^	61	60.7	330	16.4	< *0.001*	0.13	0.07, 0.23
Undetermined	33		50				
Total	744	43.6	3453	14.1	< *0.001*	0.21	0.18, 0.25

The number of observations and proportion of potential mosquito larval habitats containing *Anopheles* spp. larvae are presented by habitat type and size for control and intervention sites with statistical comparison using logistic regression (significant values appear in italics). A small number of habitats that were not classified into larger groups or the size was not able to be determined were not included in the above statistical comparison

The temporal patterns of observing anopheline pupae and late instar larvae are presented for treated and untreated sites in Fig. 3. In the first two weeks of the study, the two untreated sites showed an absence of pupae. However, by week three, pupae were present at 29.4% of the identified larval habitats in those sites, peaking at 50% by week 11. Conversely, the treated sites started with an average of 22.9% of habitats containing pupae during the first study week, decreased the following week to 3.2% and remained below 12% for the remainder of the study. The presence of late instar larvae in the untreated sites showed a steep increase following the onset of rains, from 10% in the first week of May to a peak of 80% in July and remained elevated through October. In late October, the decision to treat all construction sites was made due to ethical concerns, given the clear impact of LSM at treatment sites compared to control sites. A week after treatment, the prevalence of pupae and late instar larvae dropped to 0% and < 15%, respectively, and remained low until the end of the study period in late December. By contrast, the prevalence of late instars in the treated sites was high at baseline (43.75%) and showed a sustained decrease thereafter. After week 25, when all sites had received treatment, the prevalence of pupae and late *Anopheles* was no longer significantly different between sites.

### Comparison of mosquito density in communities adjacent to control and intervention sites

All mosquitoes were morphologically identified by trained entomologists at the Bioko Island Malaria Control Insectary. *Anopheles* spp. represented the most frequently collected adult mosquito during HLC, with 1,173 (67%) adults collected, followed by *Culex* spp. (20%), and *Aedes* spp. (13%). The total number of HLC collection events that occurred during this study was equal to 504, with 72 individuals (48 at Bti treatment sites, 24 at control sites) collecting once per month over a period of seven months. Excluding observations after October, when all sites had been treated, the average HBR was significantly lower in treated (1.5 b/p/h; range 0 to 5 b/p/h) compared to untreated sites (2.4 b/p/h; range 1 to 12 b/p/h; p < 0.001), with an estimated 38% reduction in HBR in treated sites (IRR: 0.62, 95% CI IRR: 0.55, 0.69). The mean HBR near the untreated sites increased from 1 b/p/h at week nine to 3.8 b/p/h by the end of October and rapidly decreased after applying larvicide. Near the treated sites, the mean HBR decreased after the first application from 2.3 b/p/h at week two to 1.4 b/p/h in the following collection at week seven and did not exceed 1.5 b/p/h for the remainder of the study period.

## Discussion

This case study documented the impact of LSM using Bti to treat anopheline larval habitats around construction projects. It was an observational study motivated by operational research to inform interventions in which, for a total of 30 weeks, 11 construction sites were monitored that had altered the local landscape and generated water collections suitable for mosquito production. Nine of these construction sites received treatment with Bti, and two were left untreated for comparison. All construction sites generated considerable potential larval habitats (mean of 382 per site). In the two construction sites where these habitats were left untreated, 43.6% (324/744 habitats) had late instar larvae, and 18% (133/744 habitats) had pupae. Moreover, HLC in communities near these construction sites showed that most adult mosquitoes collected (67%) were *Anopheles* spp. Treatment with Bti effectively reduced detections of immature mosquitoes within the aquatic habitat. In addition, the odds of finding immature stages in treated sites was significantly lower than in untreated sites, with an estimated reduction of 85% (OR: 0.15; 95% CI OR: 0.12, 0.2) of the prevalence of pupae and 79% (OR: 0.21; 95% CI OR: 0.18, 0.25) of the prevalence of late instar larvae. HBR was also significantly lower in neighbouring communities of treated sites than untreated ones (IRR: 0.62, 95% CI IRR: 0.55, 0.69). A natural decrease in both larvae and pupae observations was observed in later months across all sites, which is likely an artifact of construction progress, as sites came closer to completion.

This study is subject to limitations inherent to the constraints in study design. First, operational priorities determined that the design could not be experimental. For example, the reason for treating most sites was the high mosquito productivity observed in the aquatic habitats at baseline, which forced prioritizing of the study towards an operational endpoint rather than an experimental one. This brought about an obvious limitation in that the number of treated and untreated sites was severely imbalanced and could not be randomized in intervention and control arms. Second, each construction site varied in size, impacting how many sites were created during construction activities. This is relevant because larviciding will vary based on how many potential larval sites are present and how much larvicide needs to be applied. Third, the study only stretched from May to December, which might skew data based on seasonality, with weekly rainfall data not capturing site specific precipitation rates. Furthermore, various locations on the island may be naturally predisposed to larger populations of *Anopheles* spp. which must be considered during the planning phase of future studies. Finally, future LSM studies on Bioko Island should monitor malaria infections in surrounding communities participating in LSM initiatives to better quantify the impact LSM has on incidence in this setting.

Larval source management using Bti is a proven intervention to reduce larval densities in treated aquatic habitats. However, the study draws attention to the potential impacts of urban development that could pose onerous challenges to malaria control programs and proposes that the Bti application is an effective intervention to treat larval habitats around construction sites actively. Only a handful of studies have reported the impact of urban construction on the generation of anopheline larval sites [15–18]. However, this problem is particularly relevant on Bioko Island, where urbanization is increasing at a rapid pace with important consequences on the local vector mosquito ecology. For example, we recently reported on a malaria outbreak in the district of Riaba, where urban development had caused major alterations to the landscape, which, together with substantial increases in rainfall, could have explained the significant increase in *An. coluzzii* and *An. melas* mosquito densities and HBR driving the surge in cases [5]. Similar phenomena could threaten other areas in sub-Saharan Africa, where urbanization trends are expected to increase steeply in the coming decades [19]. Moreover, the potential spread of *An. stephensi* throughout the continent could aggravate this threat [20, 21]. Larval source management with Bti appears to be a cost-effective intervention that disrupts mosquito ecology and decreases the number of mature mosquitoes in an area when done correctly [22–24], with the added advantage of targeting all mosquito species, which may reduce the risk of other circulating vector-borne diseases. However, integrating vector surveillance with construction activity and creating a strategy for vector-borne disease control requires multi-sectoral coordination and cooperation with local governmental agencies and construction companies [25–27]. Previous work has suggested the potential feasibility of community participation in LSM on Bioko Island, that might also be applicable to managing temporary larval habitats adjacent to construction sites [28].

## Conclusion

This study suggests that LSM should be incorporated into vector-control strategies targeting large-scale construction projects in malaria-endemic communities. Stakeholders and construction companies could potentially cover the costs associated with entomological monitoring and the deployment of LSM during their operation to suppress vector populations surrounding their project sites. However, such strategies would require buy-in from leadership and would likely require a change in government policies to demand such interventions during permit processing. At the very least, multi-sectoral coordination needs to be in place to advise local health authorities of potential vector-borne disease risks associated with the proliferation of temporary larval habitats that are common by-products of construction activities.

## Supplementary Information

**Additional file 1: Fig. S1.** LSM team working at an active construction site.

**Additional file 2: Fig. S2.** Completed construction project.

## Data Availability

The datasets generated for this study will be shared upon reasonable request.

## Abbreviations

Bti: *Bacillus thuringiensis israelensis*
HBR: Human biting rate
HLC: Human landing catches
LSM: Larval source management
