# Supplementary figures and images for: The need for larval source management accompanying urban development projects in malaria endemic areas: a case study on Bioko Island
Source: Malar J. 2022 Nov 14;21:328. doi: 10.1186/s12936-022-04362-9 (PMC9664620; doi:10.1186/s12936-022-04362-9)

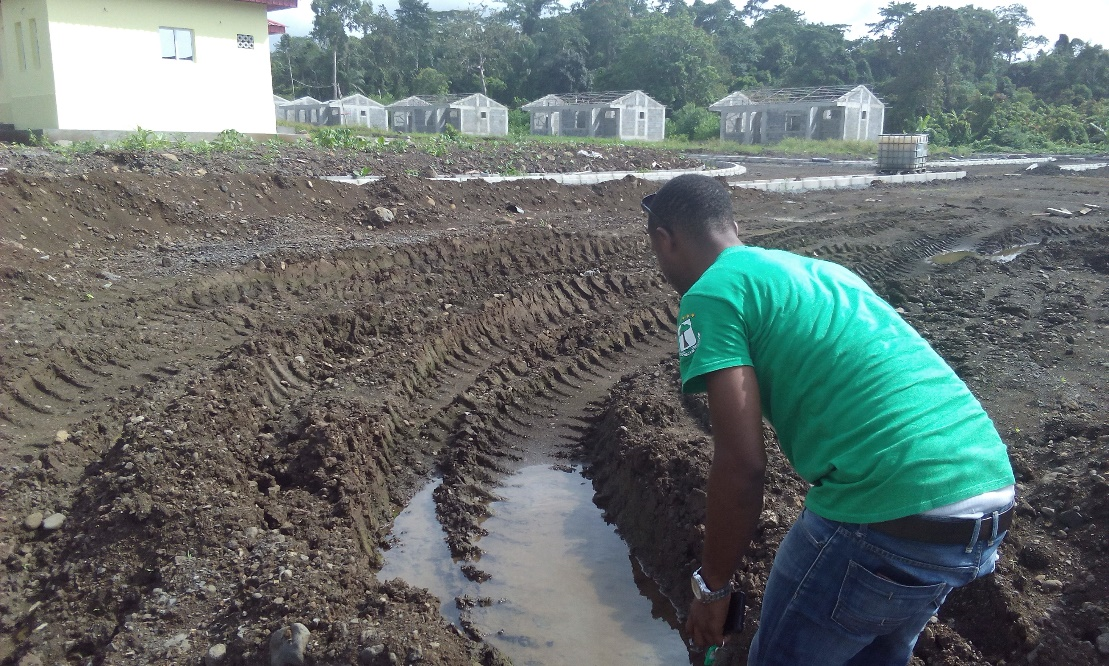

Supplement: Supplementary file 1 — Additional file 1: Fig. S1. LSM team working at an active construction site. [file 12936_2022_4362_MOESM1_ESM.tif]

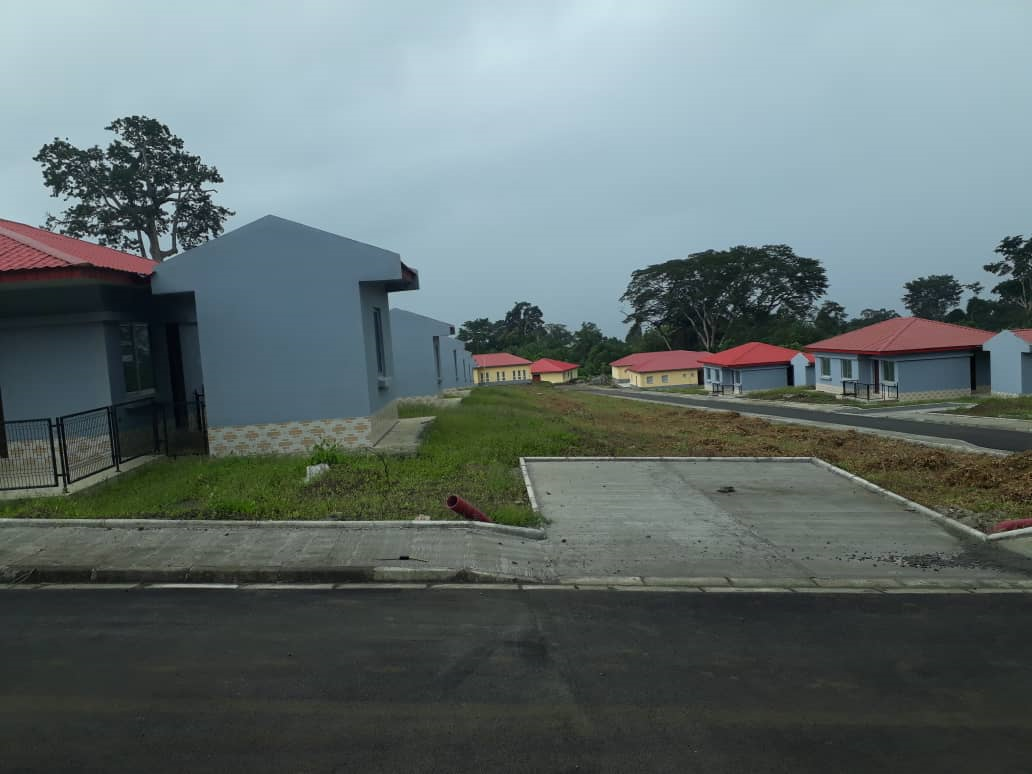

Supplement: Supplementary file 2 — Additional file 2: Fig. S2. Completed construction project. [file 12936_2022_4362_MOESM2_ESM.tif]
